# Statistical Consistency in Artificial Intelligence-Assisted Computations: A Comparison of SPSS 31.0 and GPT-5.5

**DOI:** 10.7759/cureus.111207

**Published:** 2026-06-20

**Authors:** Frederick F Strale, Rachael M German, VeraLucia Mendes-Kramer, Steven B Hammer, Robert R Ehrman, Robert L Sherwin

**Affiliations:** 1 Eugene Applebaum College of Pharmacy and Health Sciences/Applied Health Sciences, Wayne State University, Detroit, USA; 2 Mortuary Science, Wayne State University Eugene Applebaum College of Pharmacy and Health Sciences, Detroit, USA; 3 Medical and Exercise Physiology, Indian River State College, Fort Pierce, USA; 4 Emergency Medicine, Wayne State University School of Medicine, Detroit, USA

**Keywords:** ai-assisted statistics, alignment, chatgpt, ibm spss, large language models

## Abstract

Background

Artificial intelligence (AI), particularly large language models (LLMs) such as ChatGPT, is increasingly being used for statistical interpretation and research support. While traditional statistical software such as IBM SPSS remains the gold standard for transparent and reproducible analyses, concerns persist regarding the accuracy, consistency, and reproducibility of LLM-generated statistical outputs. Given the continuous evolution of LLMs, replication studies are needed to evaluate whether newer models demonstrate improved statistical reliability and alignment with established analytic standards.

Methodology

In total, 14 statistical procedures were applied to real datasets that previously generated peer‑reviewed, published scientific articles. The analyses encompassed descriptive statistics, Pearson product-moment correlation coefficient (Pearson r), multiple correlation using Pearson r, Spearman’s rho, simple linear regression, one-sample and paired t‑tests, two independent‑sample t‑tests, multiple linear regression, one-way analysis of variance (ANOVA), repeated‑measures ANOVA, two‑way (factorial) ANOVA, and multivariate analysis of variance (MANOVA). Datasets were collected within a systematically defined timeframe (2012-2023), ensuring temporal consistency and representativeness. All analyses were executed by copying and pasting prompts into GPT-5.5, accompanied by the corresponding SPSS 31.0 variable names copied and pasted directly from the original SPSS datasets.

Results

Results demonstrated concordance between SPSS 31.0 and GPT-5.5 across most descriptive and inferential statistical procedures, although notable discrepancies did occur. Identical or near-identical findings were observed for descriptive statistics, Pearson correlations, one-sample t-tests, paired t-tests, two independent-sample t-tests, simple and multiple linear regression, and one-way ANOVA. Correlation coefficients, effect sizes, confidence intervals, and significance values were mostly consistent across platforms. Discrepancies emerged in selected nonparametric analyses, factorial ANOVA, MANOVA, and repeated-measures ANOVA procedures. Despite these computational differences, the overall substantive interpretations and statistical conclusions remained largely consistent between SPSS 31.0 and GPT-5.5, but not perfectly. These findings suggest GPT-5.5 demonstrates some statistical consistency with traditional statistical software for many common analytic procedures, while more advanced multivariate analyses may still require independent verification using established statistical platforms.

Conclusions

GPT-5.5 demonstrated considerable agreement with SPSS 31.0 across many commonly used statistical procedures, including descriptive statistics, correlations, regression analyses, t-tests, and one-way ANOVA. However, more notable discrepancies emerged in several analyses, particularly more complex procedures such as factorial ANOVA, MANOVA, and repeated-measures ANOVA. These findings suggest that GPT-5.5 may serve as a useful adjunct for statistical interpretation and exploratory research, but AI-generated statistical outputs should be interpreted with caution. Validated statistical software and appropriate methodological oversight remain essential for confirmatory analyses and research involving clinical decision-making.

## Introduction

Artificial intelligence (AI) has become increasingly integrated into scientific research workflows, particularly in areas requiring rapid data interpretation, statistical computation, and methodological decision-making [1,2]. Large language models (LLMs) such as ChatGPT [3] have demonstrated the ability to generate statistical output, interpret analytic procedures, and provide narrative summaries that resemble the work of trained analysts [2,4]. As these systems continue to evolve, researchers and clinicians are increasingly asking whether AI‑generated statistical results can be considered accurate, reproducible, and trustworthy enough to support biomedical research and evidence‑based practice [1,5].

Traditional statistical software packages, most notably IBM SPSS [6], and others (i.e., SAS, R, STATA, etc.) remain the gold standard for conducting hypothesis tests, regression modeling, and multifactorial and multivariate analyses in the health sciences [2]. These platforms provide transparent algorithms, stable versioning, and well‑documented procedures that support reproducibility [7]. In contrast, LLMs are dynamic systems whose internal parameters, training data, and reasoning strategies change frequently [8]. This creates a unique challenge: while LLMs may appear to perform statistical tasks competently, their outputs may vary across versions, updates, or even repeated prompts [2]. As a result, empirical evaluation of LLM‑based statistical performance is essential [2,9].

Strale et al. [10] conducted one of the earliest systematic comparisons of SPSS output with results generated by ChatGPT‑4 and the o3 mini models. Their study demonstrated high concordance for descriptive statistics and basic inferential tests but also identified notable discrepancies in factorial and multivariate modeling, e.g., factorial analysis of variance (ANOVA) and multivariate analysis of variance (MANOVA). These findings raised important questions about the reliability of LLMs for advanced statistical work and highlighted the need for ongoing monitoring as models evolve [2,9-11].

Because LLMs undergo continuous updates, replication is not merely a scientific ideal but a practical necessity [7,12]. Unlike traditional software, where version changes are infrequent and well documented, LLMs may experience “model drift,” in which performance characteristics shift over time due to architectural changes, reinforcement learning updates, or training data modifications [8]. Therefore, replication studies serve a dual purpose, as they verify whether earlier findings remain valid and assess whether newer model versions demonstrate improved accuracy, stability, and alignment with established statistical standards [2,7,9].

Study objective and rationale

The present study replicates and extends the research of Strale et al. [10] by investigating and re‑evaluating SPSS 31.0 results against the current GPT-5.5 model using real datasets, identical prompts, and parallel analytic procedures [2,9]. By examining simple, intermediate, and advanced statistical tests, this replication aims to determine whether previously observed alignment with simpler statistical protocols and discrepancies within multifactorial and multivariate models, namely, factorial ANOVA and MANOVA, persist; whether model performance has improved; and whether LLM‑based statistical tools can be considered reliable for biomedical research applications [1,2]. Through this updated analysis, we seek to clarify the evolving role of AI in quantitative research and to inform best practices for integrating LLM‑generated statistical output into scientific workflows [1,4].

## Materials and methods

Data collection

This study was conducted from May 1, 2026, through May 19, 2026, drawing upon and critically evaluating quantitative datasets derived from peer-reviewed, published research conducted across multiple data-collection sites [13-15]. All datasets were originally generated within a systematically defined timeframe (2012-2023), ensuring temporal consistency and representativeness. The included studies primarily investigated health and biologically oriented variables, encompassing descriptive characteristics, intervariable associations, and univariate and multivariate group-based differences relevant to evidence-based therapeutic and clinical interventions. Each dataset clearly delineated coded independent variables suitable for group comparisons, such as treatment conditions, intervention phases, and groupings. Dependent variables reflected quantifiable physiological or behavioral outcomes. The repeated-measures structure present in one dataset further enabled within-subject analyses across multiple time points, increasing statistical power and supporting the evaluation of longitudinal treatment effects [15].

AI-assisted analyses were conducted using ChatGPT Plus (OpenAI), powered by the GPT-5.5 model, accessed from May 1, 2026, through May 19, 2026. The analytic protocols included descriptive statistics, simple and multiple Pearson and Spearman correlations, one-sample t-tests, paired t-tests, two independent-samples t-tests, simple and multiple linear regression, one-way ANOVA, two-way (factorial) ANOVA, MANOVA, and repeated-measures ANOVA.

Each statistical procedure was executed in SPSS 31.0 according to established analytic protocols and best-practice guidelines [6]. All hypothesis tests were two-tailed with an alpha level of 0.05. Both the SPSS 31.0 reference analyses and the corresponding GPT-5.5 outputs were generated using the standard type III sums of squares framework and the default contrast-coding specifications for all ANOVA and MANOVA procedures. Each analysis incorporated the relevant assumption tests, including evaluations of homogeneity of variance and other procedure-specific assumptions. For repeated-measures ANOVA, Mauchly’s test of sphericity was examined, and when appropriate, Greenhouse-Geisser, Huynh-Feldt, and lower-bound corrections were reported.

Missing data in SPSS were handled using the default setting of “Exclude cases analysis by analysis,” and equivalent procedures were mirrored in GPT-5.5. Consequently, observed differences in sample size across analyses reflect different variables, rather than differences in missing-data handling between platforms.

All datasets and corresponding statistical outputs were housed within SPSS 31.0 and served as the basis for direct comparisons between SPSS 31.0-generated results and outputs produced by GPT-5.5 [3,6]. The datasets were accessed within SPSS 31.0, and the corresponding SPSS 31.0 file names (e.g., Blood Pressure Data Complete Set n=236(1).sav, Lou Kramer’s Dissertation Data #2.sav, and Stroke & Heart Attack Data (1).sav) were copied and pasted into GPT-5.5 together with the appropriate prompts to perform parallel statistical analyses. No prompt repetition was utilized; rather, a single GPT-5.5 prompt was used for each individual statistical analysis. Analyses were also repeated to assess output consistency. The resulting outputs from both platforms were subsequently compared for consistency, reproducibility, and statistical concordance.

To preserve methodological integrity, identical datasets, without any post-import modifications, transformations, or preprocessing adjustments, were used for both SPSS 31.0 and GPT-5.5 analyses. This ensured that both platforms operated under equivalent analytic conditions [13-15]. Data accuracy was supported through prior logic checks, variable-type verification, and consistent formatting during the original SPSS 31.0 data-entry and cleaning processes. Numeric and categorical variables were maintained in their appropriate formats, and clear variable labeling facilitated accurate interpretation of results. Because all error-detection and data-validation procedures had been completed during the initial SPSS 31.0 workflow, the datasets used in this study were already verified as high quality and analytically sound. Consequently, the comparative evaluation of SPSS 31.0 and GPT-5.5 outputs rests on a solid empirical foundation, strengthening the credibility and interpretability of the findings [13-15].

With respect to reproducibility, it is important to acknowledge a current limitation in AI-methods reporting. The GPT-5.5 interface does not provide end users with model-specific technical identifiers such as build numbers, weight versions, checksums, or API revision numbers. As a result, documenting the subscription tier, model designation, and date range of access represents the highest level of methodological transparency presently achievable for studies utilizing the consumer-facing ChatGPT Plus platform. Consistent with emerging recommendations for reporting AI-assisted research methods, these details have been provided to facilitate future replication and methodological comparison studies while recognizing the evolving nature of LLM deployment and versioning.

Data prompts for GPT-5.5

The analyses listed in Table 1 were conducted by copying and pasting the text prompts below and the relevant SPSS 31.0 file name from real datasets [13-15], which were copied from the SPSS 31.0 dataset and pasted into GPT Plus 5.5.

**Table 1:** Test statistic with GPT-5.5 data prompts.

Test statistic	Data prompts for GPT-5.5
Descriptive statistics	Please compute the mean, standard deviation, median, mode, Shapiro-Wilk and Kolmogorov-Smirnov Test for Normality with p-values for the variable HR_pre_Sup in this “Blood Pressure Data Complete Set n=236(1).sav” SPSS dataset
Pearson correlations (r)	Please compute the mean, standard deviation and Pearson r (with confidence interval) with p-values for the variables Age and HR_pre_Sup in this “Lou Kramer’s Dissertation Data #2.sav” SPSS dataset
Multiple correlations (r)	Please compute Pearson r (with confidence interval) with p-values for the variables Q1_1 vs Q1_2, Q1.1 vs Q1_3, Q1_1 vs Q1_4, Q1_1 vs Q1_5, Q1_1 vs Q1_6 in this “Lou Kramer’s Dissertation Data #2.sav” SPSS dataset
Spearman’s rho	Please produce a Spearmen’s Rho Correlation from this SPSS dataset with the variables: AGE and PACE from this “Blood Pressure Data Complete Set n 236(1).sav” SPSS dataset. Please include confidence intervals and p-values
Spearman’s rho multiple correlations	Please produce a Spearman’s rho Correlation matrix with the variables Q1_1 vs Q1_2, Q1_1 vs Q1_3, Q1_1 vs Q1_4, Q1_1 vs Q1_5., Q1_1 vs Q1_6, in this “Lou Kramer’s Dissertation Data #2.sav” SPSS dataset. Please include confidence intervals and p-values (two-tailed)
Simple linear regression	Please run a simple linear regression with these SPSS variables from the file, “Blood Pressure Data Complete Set n=236 (1).sav.” with SUP_SYS_pre as the predictor (independent)variable and SUP_SYS_post as the outcome (dependent) variable. Please compute R, R2, adjusted R2, Standard Error of the Estimate, F, p value, t, p-value, B, Standard Error, and Beta
One-sample t-test	Please run a One Sample t-test with mu=128, with the STD_SYS_PRE variable from the SPSS file, “Blood Pressure Data Complete Set n=236(1).sav.” Please compute the n, mean, standard deviation, standard error of the mean, t, df, p-value, Mean difference, 95% confidence interval, Cohen’s d, and the 95% confidence interval for Cohen’s d
Paired t-test	Please run a Paired t-test, with this STD_SYS_PRE variable as the Pretest and the STD_SYS_POST variable as the Posttest from the file, “Blood Pressure Data Complete Set n=236(1).sav” in SPSS. Please compute the n, Pretest Mean, Posttest Mean, standard deviation, standard error of the mean, t, df, p-value, Mean difference, 95% confidence interval, Cohen’s d, and the 95% confidence interval for Cohen’s d
Two independent sample t-test	Please run a Two Independent Sample t-test, with Gender as the grouping variable (independent variable) and HR Pre Race Supine as the dependent variable with this “Blood Pressure Data Complete Set n=236(1).sav” SPSS dataset. Please compute the n, the Group 1 (Male) and Group 2 (female) Means, standard deviations, standard errors of the means, t, df, p-value, Mean difference, 95% confidence interval, Cohen’s d, and the 95% confidence interval for Cohen’s d
Multiple linear regression	Please run a multiple linear regression with these SPSS variables from the dataset, “Blood Pressure Data Complete Set n=236(1).sav,” Age, HR_PRE_SUP,, and SYS_PRE_SUP as the predictor (independent) variables and SUP_SYS_POST as the outcome (dependent) variable. Please compute R, R2, adjusted R2, Standard Error of the Estimate, F, p-value, t, p-value, B, Standard Error, and Beta
One-way analysis of variance (ANOVA)	Please run a One-Way Analysis of Variance (ANOVA) with this SPSS dataset “Lou Kramer’s Dissertation Data #2.sav” with Group as the Grouping (independent) variable with 3 groups and Q1_1 as the dependent variable. Please compute the ANOVA Table with Between and Within Groups Sums of Squares and Between and Within Groups Mean Square, F, p-value, and Effect size (Eta-Squared) with CI
Repeated-measures ANOVA	Please run a Repeated Measures ANOVA on these SPSS variables in the file, “Stroke & Heart Attack Data (1).sav,” WellBeingScoreAtDischarge, WellBeingAt3MonthsScore, WellBeingAt6MonthsScore
Two-way (factorial) ANOVA	Please run a Two-Way (Factorial) Analysis of Variance (ANOVA) with the dataset, “Blood Pressure Data Complete Set n=236(1).sav” with GENDER (2 groups) and Fram_age_Groups (12 groups) as the Grouping (independent) variables and MAP_Pre_Sup as the dependent variable. Please compute the ANOVA Table (Test of Between=Subjects Effects) with Source (Corrected Model, Intercept, Gender, Fram_age_Groups, Gender x Fram_age_Groups, Error, Total, and Corrected Total), with Type III Sum of Squares, df, Mean Square, F, p-values. and Partial Eta-Squared. Also, please do an R squared and Adjusted R-squared computation
Multivariate analysis of variance (MANOVA)	Please run a Multivariate Analysis of Variance (MANOVA) with this SPSS dataset “Lou Kramer’s Dissertation Data #2.sav” using Group as the independent (grouping) variable, and Q1_1, and Q2_2 as the dependent variables. Please run Box’s M and other tests of assumptions and report the Multivariate Tests’ relevant statistics for Pillai’s Trace, Wilks’ Lambda, Hotelling’s Trace, and Roy’s Largest Root, as well as Tests for Between Subjects Effects

Strict attention was devoted to data formatting within IBM SPSS 31.0 to ensure consistency in variable classification (e.g., numeric versus categorical variables), thereby preserving standardized input structures across all three analytical platforms. By maintaining these consistent controls, the study enabled a direct and objective comparison of outputs generated by IBM SPSS Statistics 31.0 and GPT-5.5, thereby supporting methodological accuracy and ensuring comparability of results across the analytical systems.

Simple statistical protocols

This section includes foundational statistical techniques used to summarize datasets and provide preliminary insights into measures of central tendency and variability. These procedures are essential for characterizing the distributional properties of the data before conducting more advanced inferential analyses.

Mean

The mean represents the arithmetic average of all observations within a dataset and serves as a primary measure of central tendency. Because it incorporates all values, it is sensitive to extreme scores and outliers, which may disproportionately influence the resulting estimate.

Standard Deviation

Standard deviation (SD) quantifies the degree of dispersion or spread of observations around the mean. Lower SD values indicate that data points are closely clustered around the mean, whereas higher SD values reflect greater variability within the dataset.

Median

The median represents the central value in an ordered dataset, dividing the observations into two equal halves. Unlike the mean, the median is resistant to the influence of outliers and skewed distributions, making it particularly useful when data deviate from normality.

Mode

The mode identifies the value that occurs most frequently within a dataset. It is especially informative for categorical or discrete variables and may assist in identifying multimodal distributions or recurring response patterns.

Inferential statistics protocols

These analytical methods enable researchers to evaluate hypotheses concerning relationships between variables or differences among groups, while facilitating the generalization of sample findings to broader populations.

Pearson Correlation Coefficient (r)

Pearson’s correlation coefficient (r) measures the strength and direction of the linear relationship between two continuous variables. Values range from -1 to +1, with values approaching ±1 indicating stronger linear associations. The test assumes that both variables are approximately normally distributed and measured on interval or ratio scales.

Spearman Rank-Order Correlation (Rho)

Spearman’s rho (ρ) is a non-parametric alternative to Pearson’s r and is applied when data are ordinal or when assumptions of normality are violated. This statistic evaluates monotonic relationships between variables, meaning relationships that consistently increase or decrease, without requiring linearity.

Simple Linear Regression

Simple linear regression estimates the relationship between a single independent (predictor) variable and a continuous dependent variable. The analysis provides regression coefficients that quantify the expected change in the dependent variable associated with a one-unit change in the predictor variable.

One-Sample t-Test

The one-sample t-test determines whether the mean of a single sample differs significantly from a known or hypothesized population mean. This procedure is frequently used to evaluate whether observed outcomes differ meaningfully from an expected reference value or baseline.

Paired t-Test

The paired sample t-test compares two related means, such as repeated measurements obtained from the same participants before and after an intervention. By accounting for within-subject variability, the test is well suited for detecting temporal or condition-related changes in repeated-measures designs.

Two Independent-Sample t-Test

The independent-samples t-test evaluates whether the means of two unrelated groups differ significantly. The procedure assumes approximate normality of the data and homogeneity of variances between groups.

Advanced statistical protocols

This section encompasses multivariate and factorial analytical approaches capable of modeling more complex relationships among variables. These methods are particularly advantageous when examining multiple predictors, multiple dependent variables, or interaction effects.

Multiple Linear Regression

Multiple linear regression extends simple regression by examining the relationship between one continuous dependent variable and two or more independent variables. This method permits evaluation of the unique contribution of each predictor while statistically controlling for the influence of other variables, thereby facilitating the assessment of confounding and mediating effects.

One-Way Analysis of Variance

One-way ANOVA tests whether statistically significant differences exist among the means of three or more independent groups. The method compares between-group variability relative to within-group variability and assumes normality and homogeneity of variances across groups.

Repeated-Measures Analysis of Variance

Repeated-measures ANOVA analyzes data collected from the same participants across multiple time points or experimental conditions. The technique accounts for correlations among repeated observations and determines whether statistically significant mean differences occur over time or across conditions.

Two-Way (Factorial) Analysis of Variance

Two-way (factorial) ANOVA evaluates both the independent and interactive effects of two categorical independent variables on a single continuous dependent variable. In addition to assessing main effects, the analysis examines interaction effects, indicating whether the effect of one factor depends on the level of the second factor.

Multivariate Analysis of Variance

MANOVA extends ANOVA to situations involving two or more dependent variables. The procedure evaluates whether group mean vectors differ across a combined set of dependent variables while accounting for intercorrelations among outcomes. MANOVA offers a more comprehensive assessment of group differences and may reduce the likelihood of Type I error when multiple dependent measures are analyzed simultaneously.

Ethical approval

The research associated with the SPSS 31.0 analytical dataset, “Blood Pressure Data Complete Set n=236(1).sav,” received approval from the Institutional Review Board (IRB) at Indian River State College (IRSC) in Fort Pierce, Florida. Written informed consent was obtained from all participants before race participation and study enrollment [13].

Blood pressure data were collected over a five-year period at multiple ultramarathon events across the state of Florida, including the Wild Sebastian 100 (2013 and 2014 spring/fall races), SkyDive Ultra 2016, Fort Clinch 2016, Keys 100 (2016-2018), and Saint Sebastian 100 (2017-2018). Participants were recruited through email invitations distributed to registered runners and through announcements made during pre-race meetings held the day before each event. No participants were excluded from the study [13].

The ultramarathon events ranged in distance from 50 to 240 km and were conducted during different seasons of the year. Race environments also varied considerably, with terrain ranging from entirely paved courses to sandy trail surfaces [13].

The research associated with the SPSS 31.0 analytical dataset, “Lou Kramer’s Dissertation Data #2.sav,” was conducted in accordance with established ethical standards for human participant research and received approval from the IRB at Wayne State University in Detroit, Michigan (approval number: IRB-23-01-5400; date: March 21, 2023). Before participation, all individuals were fully informed of the nature, purpose, and procedures of the study, and written informed consent was obtained from each participant before enrollment and data collection commenced [14].

This Dissertation research adhered to institutional and ethical guidelines designed to protect participant rights, confidentiality, and privacy throughout the research process. Participation was voluntary, and all participants were afforded the opportunity to decline or withdraw from the study at any time without penalty. Data included in the SPSS 31.0 analytical dataset were collected and maintained in a manner consistent with accepted standards for academic and scientific research involving human subjects [14].

The research associated with the SPSS 31.0 analytical dataset, “Stroke & Heart Attack Data (1).sav” was obtained from Statistics for the Health Sciences: A Non‑Mathematical Introduction [15], where it is provided as an instructional example. Only the underlying numerical values were used; no tables, figures, or textual material from the textbook were reproduced. Use of the data for research purposes is permitted under the fair dealing provisions referenced in the textbook’s copyright notice [15].

## Results

Simple statistics

Descriptive Statistics

Per Table 2, the results from IBM SPSS Statistics 31.0 and GPT-5.5 demonstrated near-perfect concordance across all descriptive statistics and normality coefficients for the variable HR_pre_Sup. The mean, SD, median, mode, Shapiro-Wilk statistic, and Kolmogorov-Smirnov statistic were identical between the two analyses, indicating that both platforms analyzed the same underlying data structure correctly.

**Table 2:** Descriptive statistics. Variable = pretest heart rate supine (HR_pre_Sup). SD = standard deviation

Model	Mean	SD	Median	Mode	Shapiro-Wilk	Kolmogorov-Smirnov	Shapiro-Wilk p-value	Kolmogorov-Smirnov p-value
SPSS 31.0	62.83	11.39	62	55, 61	0.952	0.077	0.003	<0.001
GPT-5.5	62.83	11.39	62	55, 61	0.952	0.077	<0.001	0.133

The only discrepancy occurred in the reported p-values for the normality tests. SPSS 31.0 reported a Shapiro-Wilk p-value of 0.003, whereas ChatGPT reported a p-value <0.001. SPSS reported a Kolmogorov-Smirnov p-value <0.001, whereas ChatGPT reported a p-value of 0.133.

The Shapiro-Wilk discrepancy is largely interpretive rather than substantive. Both values indicate statistical significance (p < 0.05) and therefore lead to the same conclusion that the distribution deviated somewhat from perfect normality. The difference likely reflects rounding, precision thresholds, or differing approximations used by the underlying computational libraries.

The larger discrepancy occurred for the Kolmogorov-Smirnov test. This difference most likely reflects methodological differences in how the test was implemented rather than a disagreement in the raw data itself. IBM SPSS Statistics 31.0 typically applies the Lilliefors correction for normality testing, whereas GPT-5.5’s statistical backend may have used a standard one-sample Kolmogorov-Smirnov test without the same correction procedure. Because the Kolmogorov-Smirnov test is highly sensitive to implementation details, sample-size adjustments, and parameter estimation methods, different software packages can produce different p-values even when the test statistic is identical. Like any discrepancy with p-values, this discrepancy (SPSS: Kolmogorov-Smirnov p < 0.001 vs. ChatGPT: p = 0.133) should be interpreted with caution.

The substantive interpretation remains largely unchanged. The descriptive statistics were mostly identical, and both analyses suggested that the variable, HR_pre_Sup, was reasonably close to normality for practical parametric analysis purposes, particularly given the sample size (n = 227).

Pearson Correlations

Table 3 indicates that the results from IBM SPSS Statistics 31.0 and ChatGPT Plus-5.5 demonstrated complete alignment. Specifically, the means and SDs were identical across both platforms. The Pearson correlation coefficients were identical. The 95% confidence intervals matched exactly, and the two-tailed p-values were identical. These findings indicate that GPT-5.5, in this instance, can reproduce SPSS 31.0 correlation results with essentially exact agreement.

**Table 3:** Pearson correlations (r). X = Q1_1, Y = Q1_2. r (95%CI) = Pearson r with the 95% confidence interval. Pearson product-moment correlation coefficient (Pearson r) was used as the statistical test to compute the p-values for this table.

Model	Mean	SD	r (95%CI)	P-value (two-tailed)
SPSS 31.0				
X	4.57	0.73	0.663 (0.431, 0.812)	<0.001
Y	4.38	0.98	–	–
GPT-5.5				
X	4.57	0.73	0.663 (0.431, 0.812)	<0.001
Y	4.38	0.98	–	–

Multiple Correlations

Table 4 presents the results from SPSS 31.0 and GPT-5.5, which demonstrated extremely strong concordance across the Pearson correlation analyses. The Pearson r coefficients and associated two-tailed p-values were identical across all variable comparisons, indicating complete agreement in the underlying correlation computations and statistical significance testing. Similarly, all p-values matched exactly, demonstrating identical inferential conclusions regarding statistical significance.

**Table 4:** Multiple correlations (Pearson r). 95% CI = 95% confidence interval. Variables = Q1_1, Q1_2, Q1_3, Q1_4, Q1_5, Q1_6. Pearson product-moment correlation coefficient (Pearson r) was used as the statistical test to compute the p-values for this table.

Model	Pearson r	P-value (two-tailed)	95% CI
SPSS 31.0			
Q1_1 vs. Q1_2	0.663	<0.001	0.431–0.812
Q1.1 vs. Q1_3	0.568	<0.001	0.299–0.754
Q1_1 vs. Q1_4	0.588	<0.001	0.326–0.766
Q1_1 vs. Q1_5	0.566	<0.001	0.296–0.752
Q1_1 vs. Q1_6	0.475	<0.003	0.418–0.807
GPT-5.5			
Q1_1 vs. Q1_2	0.663	<0.001	0.431–0.812
Q1.1 vs. Q1_3	0.568	<0.001	0.299–0.754
Q1_1 vs. Q1_4	0.588	<0.001	0.326–0.766
Q1_1 vs. Q1_5	0.566	<0.001	0.296–0.752
Q1_1 vs. Q1_6	0.475	<0.003	0.177–0.687

The only discrepancy occurred in the confidence interval reported for Q1_1 vs. Q1_6: SPSS: 0.418 to 0.807, GPT-5.5: 0.177 to 0.687. Because the Pearson r and p-values were identical, this isolated confidence interval difference most likely reflects differences in confidence interval estimation methodology, such as Fisher z-transformation implementation, rounding precision, or software-specific interval correction procedures. Nonetheless, any discrepancies should raise concern.

Importantly, both confidence intervals excluded zero and therefore supported the same substantive conclusion, a statistically significant positive association between Q1_1 and Q1_6. Overall, the analyses demonstrated near-perfect alignment and strong methodological concordance between SPSS 31.0 and GPT-5.5.

*Spearman*’*s Rho*

Table 5, illustrating SPSS 31.0 vs. GPT-5.5 results, demonstrates strong overall concordance, although the analyses were not perfectly identical. Importantly, both platforms produced the same substantive conclusion: there was a small statistically significant negative association between AGE and PACE. Several results were either identical or nearly identical. The SDs matched exactly. The mean for Y(PACE) was identical, and the Spearman’s rho coefficients were extremely close: SPSS: rho = -0.157, rho = -0.153.

**Table 5:** Spearman’s rho. X = age; Y = pace; SD = standard deviation; 95% CI = 95% confidence interval. Spearman’s rho was used as the statistical test to compute the p-values for this table.

Model	Mean	SD	Spearman’s rho (95% CI)	P-value (two-tailed)
SPSS 31.0				
X	43.68	10.87	-0.157 (-0.283, -0.026)	0.016
Y	3.79	1.32	–	–
GPT-5.5				
X	43.68	10.87	-0.153 (-0.278, -0.023)	0.021
Y	3.79	1.32	–	–

The confidence intervals were also highly similar but not identical: SPSS: (−0.283, −0.026), ChatGPT: (−0.278, −0.023). These small differences are well within the range expected from minor computational and methodological variation between statistical platforms. The primary discrepancy occurred in the two-tailed p-value: SPSS: p = 0.016, GPT-5.5: p = 0.021. As with any statistical discrepancies, this should raise concern.

This slight difference most likely reflects differences in handling of tied ranks, exact versus asymptotic significance estimation, confidence interval methodology, or floating-point precision and rounding. Because Spearman’s rho is rank-based, even very small differences in tie correction procedures can slightly alter the correlation estimate, standard error, and resulting p-value.

Importantly, both p-values remained below the conventional significance threshold (p < 0.05), meaning the inferential interpretation was unchanged across both analyses. Overall, the results mostly demonstrated methodological agreement between IBM SPSS Statistics and GPT-5.5, expected software-level variation in nonparametric statistical estimation.

*Spearman*’*s Rho Multiple Correlations*

Table 6 indicates that the SPSS 31.0 and GPT-5.5 results demonstrated extremely high agreement across all five Spearman’s rho analyses. The correlation coefficients (ρ) and two-tailed p-values were identical for every comparison, indicating that both systems produced the same inferential conclusions regarding the strength, direction, and statistical significance of the relationships among the variables.

**Table 6:** Spearman’s rho multiple correlations. 95% CI = 95% confidence interval. Variables = Q1_1, Q1_2, Q1_3, Q1_4, Q1_5, Q1_6 Spearman’s rho was used as the statistical test to compute the p-values for this table.

Model	Spearman’s rho	P-value (two-tailed)	95% CI
SPSS 31.0			
Q1_1 vs. Q1_2	0.613	<0.001	0.378, 0.797
Q1.1 vs. Q1_3	0.441	0.006	0.127, 0.675
Q1_1 vs. Q1_4	0.479	0.003	0.174, 0.700
Q1_1 vs. Q1_5	0.533	<0.001	0.243, 0.735
Q1_1 vs. Q1_6	0.494	0.002	0.193, 0.710
GPT-5.5			
Q1_1 vs. Q1_2	0.613	<0.001	0.386, 0.793
Q1.1 vs. Q1_3	0.441	0.006	0.137, 0.670
Q1_1 vs. Q1_4	0.479	0.003	0.183, 0.695
Q1_1 vs. Q1_5	0.533	<0.001	0.252, 0.731
Q1_1 vs. Q1_6	0.494	0.002	0.203, 0.705

The only discrepancies appear in the 95% confidence intervals, and these differences are small. These confidence interval differences (generally ranging from approximately 0.004 to 0.010) do not alter interpretation. In all cases, the intervals substantially overlap, the lower bounds remain well above zero, and the substantive conclusions remain unchanged.

The most likely explanation for these minor discrepancies is methodological variation in confidence interval estimation procedures. SPSS 31.0 may use a slightly different algorithm, approximation method, or rounding convention for Spearman’s rho confidence intervals than the procedure implemented within GPT-5.5. Common sources of such variation include Fisher z-transformation differences, bootstrap versus asymptotic estimation, continuity corrections, or internal rounding precision.

Importantly, the observed discrepancies are not evidence of analytical error. Rather, they reflect negligible computational differences that commonly occur across statistical software packages while still yielding equivalent inferential outcomes.

Overall, the results indicate concordance between SPSS 31.0 and GPT-5.5, with identical correlation coefficients and significance testing results, and only minimal, practically insignificant variation in confidence interval boundaries. Regardless, this should be a concern.

Simple Linear Regression

The results presented in Table 7 demonstrate complete concordance and perfect alignment between SPSS 31.0 and GPT-5.5 for the simple linear regression analysis. Every reported statistical value was identical across both platforms, including the correlation coefficient (R = 0.477), coefficient of determination (R² = 0.227), adjusted R² (0.222), standard error of the estimate (SEE = 12.782), F statistic (F = 41.510), regression coefficient (B = 0.453), standard error (SE = 0.07), standardized beta (β = 0.477), t-statistic (t = 6.24), and all associated p-values (<0.001). This indicated that the predictor variable, SUP_SYS_pre, significantly predicted the outcome variable, SUP_SYS_post. This exact agreement indicates that GPT-5.5 reproduced the regression computations with a level of precision equivalent to SPSS 31.0 for this dataset and model specification.

**Table 7:** Simple linear regression. SEE = standard error of the estimate; SE = standard error of the mean; β = beta coefficient. Simple linear regression was used as the statistical test to compute the p-values for this table.

Model	R	R^2^	Adjusted R^2^	SEE	F	P-value (two-tailed)	t	P-value	B	SE	β
SPSS 31.0	0.477	0.227	0.222	12.782	41.510	<0.001	6.443	<0.001	0.453	0.070	0.477
GPT-5.5	0.477	0.227	0.222	12.782	41.510	<0.001	6.443	<0.001	0.453	0.070	0.477

One-Sample t-Test

Table 8 indicates that the identical results obtained from SPSS 31.0 and GPT-5.5 have complete computational concordance between the two platforms for this one-sample t-test. This suggests that, when properly structured data and clearly defined missing values are provided, GPT-5.5 may accurately reproduce standard SPSS 31.0 inferential statistics.

**Table 8:** One-sample t-test. Variable = STD_SYS_PRE. SD = standard deviation; CI = confidence interval; SE = standard error of the mean; df = degrees of freedom; ES = effect size; 95% CI = 95% confidence interval. The one-sample t-test was used as the statistical test to compute the p-values for this table.

Model	n	Mean	SD	SE	t	df	P-value (two-tailed)	Mean difference	95% CI for mean difference	ES (Cohen’s d)	ES 95% CI
SPSS 31.0	225	131.68	15.89	1.059	3.478	224	<0.001	3.684	1.60, 5.77	0.232	0.099, 0.364
GPT-5.5	225	131.68	15.89	1.059	3.478	224	<0.001	3.684	1.60, 5.77	0.232	0.099, 0.364

The analysis showed that the mean of the variable STD_SYS_PRE (mean = 131.68, SD = 15.89) was significantly higher than the hypothesized comparison value (mu) of 128. The one-sample t-test was statistically significant (t(224) = 3.48, p < 0.001). The mean difference was 3.68 points, and the 95% confidence interval for the mean difference (1.60, 5.77) did not include zero, supporting the reliability of the effect.

The effect size was small: Cohen’s d = 0.23, with a 95% confidence interval of (0.099, 0.364), indicating that although the difference was statistically significant, the magnitude of the effect was small in practical terms. Overall, the findings suggest that the variable STD_SYS_PRE’s mean was reliably, but only slightly, higher than the reference value (mu) of 128.

Paired t-Test

As indicated in Table 9, the results presented demonstrate complete concordance between SPSS 31.0 and GPT-5.5 for the paired t-test protocol of STD_SYS_PRE and STD_SYS_POST. Every reported statistical value was identical across the two platforms, indicating perfect computational alignment and reproducibility of the analysis.

**Table 9:** Paired t-test. Variables = STD_SYS_PRE, STD_SYS_POST. SD = standard deviation; CI = confidence interval; SE = standard error of the mean; df = degrees of freedom; ES = effect size; 95% CI = 95% confidence interval. The paired t-test was used as the statistical test to compute the p-values for this table.

Model	n	Mean	SD	SE	t	df	P-value	Mean difference	95%CI	Effect size (Cohen’s d)	Effect size 95% CI
SPSS 31.0											
STD_SYS_PRE	144	130.87	15.819	1.318	14.584	143	<0.001	20.139	17.409, 22.869	1.215	0.998, 1.430
STD_SYS_POST	144	110.73	13.408	1.117	–	–	–	–	–	–	–
GPT-5.5											
STD_SYS_PRE	144	130.87	15.819	1.318	14.584	143	<0.001	20.139	17.409, 22.869	1.215	0.998, 1.430
STD_SYS_POST	144	110.73	13.408	1.117	–	–	–	–	–	–	–

This perfect agreement indicates that GPT-5.5 replicated the SPSS 31.0 computations exactly, without any observable discrepancies due to rounding, estimation procedures, or computational precision. Unlike prior analyses where very small confidence interval differences emerged, the present results demonstrate full numerical equivalence across all descriptive and inferential statistics. Overall, these findings provide strong evidence of analytic consistency and exact reproducibility between SPSS 31.0 and GPT-5.5 for this paired-samples t-test analysis.

Two Independent-Samples t-Test

Table 10 indicates that the results presented demonstrate complete agreement between SPSS 31.0 and GPT-5.5 for the two independent-samples t-test comparing males and females on the HR_pre_Sup variable. All reported descriptive and inferential statistics were identical across both platforms, indicating perfect concordance in the analysis.

**Table 10:** Two independent-samples t-test. SD = standard deviation; CI = confidence interval; SE = standard error of the mean; df = degrees of freedom; ES = effect size; 95% CI = 95% confidence interval. The two independent-samples t-test was used as the statistical test to compute the p-values for the table.

Model	n	Mean	SD	SE	t	df	P-value (two-tailed)	Mean difference	95%CI	Effect size (Cohen’s d)	Effect size 95% CI
SPSS 31.0											
Group 1-Male	141	62.48	10.591	0.892	-0.605	225	0.546	-0.943	-4.088, 2.131	0.083	-0.351, 0.186
Group 2-Female	86	63.42	12.628	1.362	–	–	–	–	–	–	–
GPT-5.5											
Group 1-Male	141	62.48	10.591	0.892	-0.605	225	0.546	-0.943	-4.088, 2.131	0.083	-0.351, 0.186
Group 2-Female	86	63.42	12.628	1.362	–	–	–	–	–	–	–

These findings indicated no statistically significant difference between males and females on the HR_pre_Sup measure. The p-value (0.546) was substantially greater than the conventional alpha level of 0.05, and the effect size was extremely small (d = 0.083), suggesting a negligible practical difference between groups.

Importantly, the perfect numerical alignment between SPSS 31.0 and GPT-5.5 indicated both systems implemented the two independent-samples t-test identically for this dataset. No discrepancies were observed in any descriptive statistic, inferential statistic, or effect size estimate. This level of agreement suggests identical computational procedures, equivalent handling of sample sizes and variance estimates, and consistent statistical precision across both platforms.

Overall, the table provides strong evidence that GPT-5.5 can reproduce the SPSS 31.0 two independent-samples t-test results with complete accuracy and consistency for this analysis.

Table 11 indicates that the results from SPSS 31.0 and GPT-5.5 demonstrated complete alignment across all multiple linear regression statistics. Every reported value matched exactly between the two platforms, including model fit statistics, regression coefficients, standard errors, standardized beta coefficients, t-statistics, and p-values.

**Table 11:** Multiple regression. SEE = standard error of the estimate; SEM = standard error of the mean. Multiple linear regression was used as the statistical test to compute the p-values for this table.

Model	R	R^2^	Adjusted R^2^	SEE	F	P-value (two-tailed)	t	P-value	B	SE	β
SPSS 31.0	0.482	0.233	0.216	12.831	14.047	<0.001	–	–	–	–	–
Age	–	–	–	–	–	–	0.939	0.349	0.106	0.113	0.072
HR_PRE_SUP	–	–	–	–	–	–	-0.068	0.946	-0.007	0.109	-0.005
SYS_PRE_SUP	–	–	–	–	–	–	6.191	<0.001	0.443	0.071	0.466
GPT-5.5	0.482	0.233	0.216	12.831	14.047	<0.001	–	–	–	–	–
Age	–	–	–	–	–	–	0.939	0.349	0.106	0.113	0.072
HR_PRE_SUP	–	–	–	–	–	–	-0.068	0.946	-0.007	0.109	-0.005
SYS_PRE_SUP	–	–	–	–	–	–	6.191	<0.001	0.443	0.071	0.466

Specifically, both analyses showed that the overall multiple regression model was statistically significant (F = 14.047, p < 0.001) and explained approximately 23.3% of the variance in SUP_SYS_POST (R² = 0.233). In both analyses, SYS_PRE_SUP emerged as the only statistically significant predictor (β = 0.466, p < 0.001), whereas AGE and HR_PRE_SUP were non-significant predictors.

One-Way Analysis of Variance

Table 12 indicates that the results in our comparison table are identical between SPSS 31.0 and GPT-5.5. The agreement across all statistics indicates that the calculations were performed correctly and consistently. The matching results demonstrated that GPT-5.5 replicated the classical ANOVA calculations exactly as SPSS 31.0 would compute them.

**Table 12:** One-way analysis of variance (ANOVA). df = degrees of freedom; ES = effect size; CI = confidence interval. One-way analysis of variance (ANOVA) was used as the statistical test to compute the p-values for this table.

Source of variation	Sums of squares	df	Mean square	F	P-value	ES (eta-squared)	ES (eta-squared) CI
SPSS 31.0							
Between groups	1.007	2	0.504	0.947	0.398	0.053	0.000, 0.207
Within groups	18.074	34	0.532	–	–	–	–
Total	19.081	36	–	–	–	–	–
GPT-5.5							
Between groups	1.007	2	0.504	0.947	0.398	0.053	0.000, 0.207
Within groups	18.074	34	0.532	–	–	–	–
Total	19.081	36	–	–	–	–	–

When identical input data are analyzed with the same statistical model and no differing options (such as Welch correction, missing-data handling, or rounding conventions), the outputs should theoretically be identical to many decimal places.

This is an important finding methodologically because it suggests that GPT-5.5 may accurately reproduce standard inferential statistics traditionally generated by dedicated statistical software such as SPSS 31.0 for this type of analysis. In applied research terms, this may strengthen confidence in the reproducibility and reliability of the GPT-5.5-generated ANOVA results relative to SPSS 31.0.

Two-Way (Factorial) Analysis of Variance

Table 13 shows that some portions of the factorial ANOVA output are perfectly aligned between SPSS 31.0 and GPT-5.5, while other portions differ substantially. These discrepancies are highly informative because they point to differences in how the categorical factors were interpreted and encoded during model construction.

**Table 13:** Two-way (factorial) analysis of variance (ANOVA). df = degrees of freedom. Two-way (factorial) analysis of variance (ANOVA) was used as the statistical test to compute the p-values for this table.

Source of variation	Type III sums of squares	df	Mean square	F	P-value	ES (partial eta-squared)	Model fit, R²	Model fit, adjusted R²
SPSS 31.0								
Corrected model	4,067.992	21	193.714	1.984	0.009	0.189	0.189	0.094
Intercept	428,920.636	1	428,920.636	4,392.827	<0.001	0.961	–	–
Gender	415.61	1	415.61	4.257	0.041	0.023	–	–
Fram-age-groups	2,054.528	11	186.775	1.913	0.04	0/105	–	–
Gender x Fram_age groups	976.539	9	108.504	1.111	0.357	0.053	–	–
Error	17,477.763	179	97.641	–	–	–	–	–
Total	1,666,953.51	201	–	–	–	–	–	–
Corrected total	21,545.755	200	–	–	–	–	–	–
GPT-5.5								
Corrected model	4,067.992	21	193.714	1.984	0.009	0.189	0.189	0.094
Intercept	21,281.152	1	21,281.152	217.953	<0.001	0.549	–	–
Gender	6.232	1	6.232	0.064	0.801	0.000	–	–
Fram-age-groups	2,555.951	12	212.996	2.181	0.017	0.128	–	–
Gender x Fram_age groups	1,128.452	12	94.045	0.963	0.482	0.061	–	–
Error	17,477.763	179	97.641	–	–	–	–	–
Total	1,666,953.51	201	–	–	–	–	–	–
Corrected total	21,545.755	200	–	–	–	–	–	–

This agreement demonstrates that both systems analyzed essentially the same total variability, the same dependent variable (MAP_Pre_Sup) was used, the same overall sample size and residual variance structure were retained, and the same overall factorial model framework was fit. The identical model fit values indicate the same global partitioning of variance. Thus, the disagreement is not due to numerical instability or arithmetic error.

There are discrepancies that raise concern. The primary source of discrepancy was different factor coding/group detection. Per Table 13, the largest discrepancies occurred in the variables Gender, Fram-age-groups, Gender × Fram_age groups, and Intercept. The critical clues are the degrees of freedom discrepancies.

This strongly suggests that GPT-5.5 interpreted the categorical variables differently from SPSS 31.0. For a factor with k groups (df = k−1). Therefore, SPSS 31.0 detected 12 age groups (df = n-1 = 11), whereas ChatGPT Plus-5.5 detected 13 levels (df = n - 1 = 12). This indicates that GPT-5.5 likely treated a missing-data code, empty category, sparse level, formatting artifact, or unlabeled value as an additional valid category. This is extremely common in imported SPSS data when value labels are not preserved, missing values are coded numerically, or unused factor levels are retained.

As to why the gender effect changed dramatically, this is a major discrepancy. The most likely explanation is that the factor structure became reparametrized differently once the age-group levels changed. In factorial ANOVA, type III sums of squares depend heavily on coding scheme, factor balance, empty cells, interaction structure, and reference category construction. Type III sums of squares are computed after adjusting for all other terms in the model.

If one platform includes an extra age-group level, collapses sparse cells differently, or handles empty Gender × Age cells differently, then the variance attributable uniquely to Gender can change dramatically. Thus, SPSS 31.0 found a small but statistically significant Gender effect, whereas GPT-5.5’s altered cell structure redistributed that variance into other terms.

As to why the interaction degrees of freedom differ, the interaction df discrepancy is especially revealing. The theoretical interaction df should be: df = (a−1)(b-1).

If Gender has two groups: a−1=1, then the SPSS interaction df = 9 implies only 10 estimable age-group contrasts remained; GPT-5.5’s interaction df = 12 implies 13 detected age levels. This strongly indicates empty/sparse cells, omitted categories, or aliased parameters in SPSS 31.0.

SPSS 31.0 automatically handles singularities and empty cells in GLM procedures, often dropping non-estimable parameters silently. GPT-5.5 likely retained all detected factor levels mathematically.

As to why the intercept is radically different, the intercept term in ANOVA depends on coding scheme, centering, reference levels, and design matrix construction. SPSS 31.0 GLM uses its own internal reference-cell coding conventions and type III estimation approach.

GPT-5.5 likely used a standard regression-based implementation without identical SPSS 31.0 contrast coding. Importantly, the intercept discrepancy is usually not scientifically meaningful, because intercepts in factorial ANOVA rarely affect substantive interpretation.

Why does the overall model still match? Even though the partitioning among effects differed, the total explained variance (R² = 0.189) remained identical. This implies that both analyses explained the same overall amount of variance, *but allocated that explained variance differently across factors.* That is exactly what happens when factor coding differs, or category structures differ.

The most likely technical explanation for the discrepancies is most likely caused by extra detected age-group categories in GPT-5.5, different missing-value handling, empty/sparse cell handling differences, different type III contrast coding, and SPSS 31.0 dropping non-estimable parameters. As always, these types of described discrepancies should have clinicians and researchers interpreting results with caution.

Table 14 indicates that SPSS 31.0 and GPT-5.5’s MANOVA results show the same overall conclusion, i.e., that there was no statistically significant multivariate effect for Group.

**Table 14:** Multivariate analysis of variance (MANOVA). df = degrees of freedom. Multivariate analysis of variance (MANOVA) was used as the statistical test to compute the p-values for this table.

Effect test statistic		Value	F	Hypothesis df	Error df	P-value	Partial eta-squared
SPSS 31.0							
Intercept	Pillai’s trace	0.961	404.155	2.000	33.000	0.000	0.961
	Wilks’ lambda	0.039	404.155	2.000	33.000	0.000	0.961
	Hotelling’s Trace	24.494	404.155	2.000	33.000	0.000	0.961
	Roy’s largest root	24.494	404.155	2.000	33.000	0.000	0.961
Group	Pillai’s trace	0.111	0.999	4.000	68.000	0.414	0.056
	Wilks’ lambda	0.892	.971^b^	4.000	66.000	0.430	0.056
	Hotelling’s trace	0.118	0.942	4.000	64.000	0.446	0.056
	Roy’s largest root	0.068	1.151^c^	2.000	34.000	0.328	0.063
GPT-5.5							
Intercept	Pillai’s trace	0.979	778.6	2.000	33.000	<0.001	0.979
	Wilks’ lambda	0.021	778.6	2.000	33.000	<0.001	0.979
	Hotelling’s trace	47.19	778.6	2.000	33.000	<0.001	0.979
	Roy’s largest root	47.19	778.6	2.000	33.000	<0.001	0.979
Group	Pillai’s trace	0.0606	0.531	4.000	68.000	0.713	0.030
	Wilks’ lambda	0.939	0.524	4.000	66.000	0.719	0.031
	Hotelling’s trace	0.065	0.527	4.000	64.000	0.717	0.032
	Roy’s largest root	0.064	1.09	2.000	34.000	0.348	0.060

As is common with different statistical software packages, such as R, SAS, STATA, Python, etc., each is approximating the same mathematical truth, but each uses different approximations, different numerical engines, different defaults, and different optimization paths. Hence, the results converge toward the same answer but sometimes do not match perfectly digit‑for‑digit.

There are discrepancies that raise concern. The largest discrepancies occur for the Intercept statistics, the magnitude of the multivariate test statistics, and the associated partial eta-squared values. For the Group effect, SPSS reported somewhat larger multivariate effects. This suggests SPSS 31.0 attributed slightly more multivariate variance to Group than GPT-5.5 did. However, both systems agreed that the multivariate effect was non-significant.

Thus, the substantive interpretation is unchanged. The discrepancies most likely arise from slightly different covariance matrix estimation, differing handling of missing cases, numerical precision/rounding, or differences in matrix decomposition algorithms used internally by SPSS 31.0 GLM versus GPT-5.5’s MANOVA implementation.

The very large differences for the Intercept are generally not substantively important. In MANOVA, the intercept tests whether the grand multivariate mean vector differs from zero, and different software implementations often parameterize this component differently. Both systems nonetheless concluded that the intercept was highly significant with extremely large effect sizes.

While the reported partial eta-squared values differed (0.056 vs. 0.031), both estimates fall within the small to medium effect range and indicate that only a modest proportion of variance in the combined dependent-variable space was attributable to Group. Although effect sizes can influence future research prioritization, it is noteworthy that each effect size estimate approaches a magnitude typically interpreted as small to medium. Consequently, the practical implications of these differences are limited, and both platforms characterize the Group effect as small to medium.

Importantly, the hypothesis df, error df, and overall inferential conclusion were highly consistent across both platforms, indicating that the discrepancies reflect implementation details rather than fundamentally different statistical conclusions.

Table 15 indicates that SPSS 31.0 and GPT-5.5 repeated-measures ANOVA results demonstrate exceptionally strong concordance and overall methodological alignment. Both systems produced identical results for: type III sums of squares, degrees of freedom, F-statistics, p-values, and partial eta-squared effect sizes across all correction methods: sphericity assumed, Greenhouse-Geisser, Huynh-Feldt, and lower-bound.

**Table 15:** Repeated-measures analysis of variance (ANOVA). df = degrees of freedom.

Source		Type III sum of squares	df	Mean square	F	P-value	Partial eta-squared
SPSS 31.0							
Time	Sphericity assumed	335.400	2	167.700	42.479	<0.001	0.521
	Greenhouse-Geisser	335.400	1.846	181.646	42.479	<0.001	0.521
	Huynh-Feldt	335.400	1.934	173.416	42.479	<0.001	0.521
	Lower-bound	335.400	1.000	335.400	42.479	<0.001	0.521
Error (time)	Sphericity assumed	307.933	78	3.948	–	–	–
	Greenhouse-Geisser	307.933	72.011	4.276	–	–	–
	Huynh-Feldt	307.933	75.429	4.082	–	–	–
	Lower-bound	307.933	39.000	7.896	–	–	–
GPT-5.5							
Time	Sphericity assumed	335.400	2	167.700	42.479	<0.001	0.521
	Greenhouse-Geisser	335.400	1.846	181.646	42.479	<0.001	0.521
	Huynh-Feldt	335.400	1.934	173.416	42.479	<0.001	0.521
	Lower-bound	335.400	1.000	335.400	42.479	<0.001	0.521
Error (time)	Sphericity assumed	307.933	78	3.948	_	_	_
	Greenhouse-Geisser	307.933	72.011	3.948	_	_	_
	Huynh-Feldt	307.933	75.429	3.948	–	–	–
	Lower-bound	307.933	39.000	3.948	–	–	–

Both SPSS 31.0 and GPT-5.5 reported a significant within-subjects effect of Time with a large effect size. The corrected degrees of freedom for the Greenhouse-Geisser and Huynh-Feldt adjustments were also identical, demonstrating that both systems handled the sphericity corrections consistently.

The only discrepancy that raises concern involved the mean square values for the corrected error (Time) rows. SPSS 31.0 recalculated the corrected mean squares separately for each sphericity adjustment, whereas GPT-5.5 retained the original uncorrected error (Time) mean square value (3.948) across the correction rows. Importantly, however, this difference did not affect the F-statistics, statistical significance, or effect size estimates, all of which remained perfectly aligned. As always, these results need to be interpreted with caution and a healthy skepticism by clinicians and researchers.

## Discussion

The present study examined the methodological consistency between SPSS 31.0 and GPT-5.5 across a broad range of statistical procedures, including descriptive statistics, correlational analyses, linear regression, t-tests, ANOVA, MANOVA, and repeated-measures ANOVA. Consistent with prior investigations into AI-assisted statistical analysis, the findings demonstrate that GPT-5.5 exhibits concordance with traditional statistical software for many fundamental and intermediate analytic procedures, while also revealing notable discrepancies in basic statistics and advanced multivariate and factorial modeling contexts.

The results suggest that contemporary LLMs are increasingly capable of reproducing conventional statistical computations with substantial accuracy when provided with clearly structured prompts and properly formatted datasets, but results must continue to be interpreted with caution. These findings support the growing body of literature indicating that AI systems may function as useful adjunctive tools in quantitative research workflows, particularly for preliminary analyses, educational purposes, and rapid interpretation tasks [1,2,4,16,17-20]. At the same time, however, the observed inconsistencies in basic and complex procedures reinforce continuing concerns regarding reproducibility, transparency, and methodological reliability in AI-generated statistical outputs [8].

A particularly important finding was the alignment, for the most part, between SPSS 31.0 and GPT-5.5 for descriptive statistics and basic inferential procedures. Mean values, SDs, medians, modes, and several normality statistics were either identical or nearly identical across platforms. Similarly, Pearson correlations and multiple Pearson correlation matrices demonstrated almost complete concordance, with identical correlation coefficients, almost identical confidence intervals, and significance levels across comparisons. These findings are noteworthy because correlational procedures represent foundational analytic operations upon which many higher-order statistical models are built. The ability of GPT-5.5 to reproduce these calculations accurately suggests that the model is capable of correctly implementing standard covariance and association computations under well-defined conditions.

The same pattern of strong agreement was observed for several parametric inferential procedures. The one-sample t-test, paired t-test, and two independent-samples t-test results produced by GPT-5.5 were essentially identical to those generated by SPSS 31.0 across all major statistical indicators, including means, SDs, standard errors, t-values, degrees of freedom, p-values, mean differences, and confidence intervals. The paired t-test results were particularly impressive, demonstrating exact agreement for sample size, mean differences, inferential significance, and effect size estimates.

From a methodological perspective, the near-perfect agreement observed in these procedures likely reflects the deterministic nature of classical parametric calculations. Procedures such as means, SDs, Pearson correlations, and t-tests rely upon relatively direct mathematical formulas that are less sensitive to implementation variability than multivariate or iterative estimation techniques. Consequently, once the model correctly interprets the prompt structure and variable relationships, the computational pathway becomes relatively straightforward. This may explain why GPT-5.5 performed particularly well on these analyses.

The simple and multiple linear regression results demonstrated excellent alignment between models, with identical values reported for R, R², adjusted R², SEE, F-statistics, predictor coefficients, and inferential significance. This degree of concordance suggests that GPT-5.5 can successfully execute and interpret standard multivariable ordinary least squares regression models when predictor structures are clearly specified.

The findings for Spearman’s rho analyses also demonstrated minor but noteworthy discrepancies. While the overall direction and magnitude of the relationships remained highly similar between SPSS 31.0 and GPT-5.5, small differences emerged in confidence intervals and p-values. For example, SPSS 31.0 produced a Spearman correlation of −0.157 with a p-value of 0.016, whereas GPT-5.5 produced a coefficient of −0.153 with a p-value of 0.021. Such differences are statistically small and unlikely to alter substantive interpretation; however, they highlight an important methodological issue regarding rank-based and nonparametric procedures. Unlike straightforward parametric statistics, nonparametric analyses may involve varying approaches to tie correction, exact versus asymptotic significance estimation, or confidence interval computation. These procedural subtleties may partially explain the slight divergence between platforms.

The ANOVA findings further illustrate the distinction between strong performance in conventional procedures and increased variability in more complex statistical modeling. The one-way ANOVA results demonstrated complete agreement between SPSS 31.0 and GPT-5.5 for sums of squares, degrees of freedom, mean squares, F-values, significance levels, and eta-squared effect sizes. This again suggests that relatively straightforward linear partitioning models are now being reproduced accurately by advanced LLM systems.

By contrast, the factorial ANOVA results revealed more substantial inconsistencies. Although the corrected model statistics were identical across platforms, important discrepancies emerged in the computation of the intercept, the main effects for gender and age group, interaction terms, and partial eta-squared estimates. SPSS 31.0 identified statistically significant effects for gender and age group, whereas GPT-5.5 failed to reproduce the gender effect and altered the degrees of freedom associated with the age-group factor. These discrepancies are methodologically meaningful because factorial ANOVA procedures involve complex variance decomposition, type III sums of squares estimation, and interaction modeling. Minor differences in coding assumptions, treatment contrasts, or missing data handling may substantially alter the resulting inferential statistics.

The MANOVA results revealed the largest discrepancies. Although both platforms agreed that the multivariate group effect was non-significant, the actual multivariate statistics differed considerably. GPT-5.5 produced substantially larger values for Pillai’s trace, Hotelling’s trace, Roy’s largest root, and associated F-statistics for the intercept term than SPSS 31.0. Partial eta-squared estimates also differed substantially across systems.

Several explanations may account for these discrepancies. First, multivariate analyses require simultaneous estimation of covariance matrices, matrix inversions, and eigenvalue decompositions, all of which are computationally more sensitive than univariate procedures. Second, SPSS 31.0 employs highly standardized algorithms with transparent matrix-based implementations, whereas LLMs may approximate procedures through probabilistic reasoning pathways rather than executing identical deterministic algorithms. Third, prompt interpretation may influence how GPT-5.5 operationalizes multivariate assumptions, including treatment of covariance homogeneity, singularity, or missing values. These issues reinforce the continuing need for caution when using AI systems for advanced multivariate analyses.

The repeated-measures ANOVA results provided an especially interesting pattern of findings. GPT-5.5 successfully reproduced all primary inferential statistics, including type III sums of squares, corrected degrees of freedom, F-values, significance levels, and partial eta-squared estimates across sphericity corrections. However, discrepancies emerged in the computation of mean square error terms under Greenhouse-Geisser and Huynh-Feldt corrections. Although these differences did not alter statistical significance or substantive interpretation, they suggest that the model may simplify certain repeated-measures covariance adjustments during estimation.

Importantly, despite these discrepancies, the overall inferential conclusions across nearly all analyses remained remarkably consistent between SPSS 31.0 and GPT-5.5. In most cases, statistically significant findings remained significant across platforms, and non-significant findings remained non-significant. This observation is critically important because it suggests that, even when minor computational variability exists, the broader substantive interpretation of the data may remain stable. From a practical standpoint, this indicates that GPT-5.5 may already possess meaningful utility as a supplementary analytic assistant for researchers, clinicians, and students, but results, again, should be interpreted with caution and a healthy skepticism.

Given this, the present findings strongly support the argument that AI-generated statistical analyses should not yet be viewed as replacements for established statistical software or expert statistical oversight. The discrepancies observed in factorial ANOVA, MANOVA, repeated-measures ANOVA, and minor discrepancies observed in multiple Spearman’s rho, Spearman’s rho, multiple correlations, and descriptive statistics computations indicate that LLMs may remain vulnerable to computational drift, prompt sensitivity, and methodological inconsistency. These findings align closely with broader concerns regarding reproducibility in AI systems [7,12,21-24]. Unlike traditional statistical packages, which rely upon transparent and stable computational architectures, LLMs operate through continuously evolving probabilistic systems whose internal mechanisms are not fully accessible to end users [8,25].

The present study also has important implications for research training and scientific workflow integration. One of the most promising uses of GPT-5.5 may lie in its ability to assist researchers with statistical interpretation, procedural explanation, syntax generation, and preliminary exploratory analysis. Indeed, the model demonstrated competence in producing organized statistical tables, effect size estimates, and narrative summaries. However, because basic and advanced procedures remain susceptible to inconsistency, researchers should continue to rely upon validated statistical software for confirmatory analyses and publication-level inference.

Limitations

Several limitations should be acknowledged when interpreting these findings. The analyses were necessarily constrained by the specific datasets and statistical procedures included in the study design; different data structures, missing‑data patterns, or non‑Gaussian distributions may produce different levels of agreement. Moreover, because GPT‑5.5 is a continuously updated system, the results observed here may not generalize to future iterations. This reality underscores the importance of ongoing replication studies as AI models evolve. Additionally, the present investigation emphasized output concordance rather than transparency of computational mechanisms. Future work should examine how LLMs internally operationalize statistical formulas and whether these internal pathways remain stable across repeated prompts or model updates.

Despite these limitations, the study revealed a notable pattern. GPT‑5.5 demonstrated concordance but not perfect alignment with SPSS 31.0 across the statistical protocols examined. This suggests that LLMs may reliably reproduce many standard statistical outputs when provided with clear prompts and well‑structured data. This level of agreement supports a cautiously optimistic view of AI‑assisted computation as a supplemental tool for researchers, educators, and clinicians.

At the same time, the discrepancies observed in some of the simpler statistics and multifactorial and multivariate procedures, particularly factorial ANOVA and MANOVA, highlight important constraints. Unlike SPSS 31.0, which applies deterministic and transparent algorithms, GPT‑5.5 relies on probabilistic reasoning shaped by training data and prompt interpretation. These differences were most evident in analyses involving interaction terms and multivariate omnibus tests, where GPT‑5.5 occasionally produced p‑values and effect sizes that diverged substantially from SPSS outputs. Instances where SPSS might report, for example, a statistically significant Kolmogorov‑Smirnov result (e.g., p = 0.001) while GPT‑5.5 returned, for example, a non‑significant Kolmogorov‑Smirnov value (e.g., p = 0.133) illustrate the potential for interpretive conflict and caution.

Such inconsistencies carry practical implications. Multifactorial and multivariate analyses often inform judgments about treatment effects, subgroup differences, and clinical risk. When AI‑generated results diverge from validated statistical software, the resulting uncertainty may influence clinical decision‑making, guideline development, or patient management. For this reason, AI‑derived statistical outputs, while increasingly capable, should be interpreted with measured caution. GPT‑5.5 and similar models may serve as valuable adjuncts for explanation, verification, or preliminary review, but they should not replace established analytic platforms when research findings may affect patient care.

Further, in the repeated-measures ANOVA, discrepancies were observed in the computation of the mean square error terms associated with the Greenhouse-Geisser and Huynh-Feldt corrections. Although these differences had no impact on statistical significance, effect size estimates, or the overall interpretation of the findings, they may reflect simplifications in the handling of covariance structure adjustments during the estimation process for repeated-measures designs.

Taken together, these findings suggest a balanced conclusion. AI‑assisted computation is emerging as a promising component of statistical workflows, yet the discrepancies identified in complex models reinforce the need for careful, cautionary verification. Researchers and clinicians should continue to rely on validated statistical software as the primary basis for inferential conclusions, particularly in contexts where patient well‑being is at stake.

Future research should extend this work by evaluating additional advanced procedures, including epidemiological modeling, logistic regression, mixed‑effects models, structural equation modeling, survival analysis, Bayesian estimation, and machine‑learning classification frameworks. Comparative investigations involving R, SAS, Stata, and Python‑based analytic environments would further clarify the relative strengths and limitations of AI‑assisted statistical systems. Longitudinal assessments of model drift across successive LLM versions will also be essential as AI technologies continue to evolve.

## Conclusions

GPT-5.5 demonstrated somewhat substantial agreement with SPSS 31.0 across a broad range of statistical procedures, particularly descriptive statistics, correlation analyses, regression models, t-tests, and one-way ANOVA. Although the overall substantive conclusions were generally consistent between platforms, discrepancies were observed in several analyses, with more pronounced differences emerging in complex procedures such as factorial ANOVA, MANOVA, and repeated-measures ANOVA. These findings suggest that GPT-5.5 and LLMs, in general, may have meaningful utility as a supplementary tool for statistical education, interpretation, and exploratory research. The observed inconsistencies indicate that AI-generated statistical outputs should be interpreted with caution by researchers and clinicians, particularly when analyses involve multifactorial, multivariate, or repeated-measures designs. As a consequence, validated statistical software and appropriate methodological oversight remain essential for confirmatory analyses, publication-quality research, and clinical decision-making. Continued validation and replication studies are warranted to further evaluate the reliability, reproducibility, and methodological consistency of AI-assisted statistical analysis as LLMs continue to evolve.
